# Phenotypic and genetic characteristics of 130 patients with mucopolysaccharidosis type II: A single-center retrospective study in China

**DOI:** 10.3389/fgene.2023.1103620

**Published:** 2023-01-13

**Authors:** Zhenjie Zhang, Mingsheng Ma, Weimin Zhang, Yu Zhou, Fengxia Yao, Lisi Zhu, Min Wei, Zhengqing Qiu

**Affiliations:** ^1^ Department of Pediatrics, Peking Union Medical College Hospital, Chinese Academy of Medical Sciences and Peking Union Medical College, Beijing, China; ^2^ Department of Genetics Laboratory, Peking Union Medical College Hospital, Chinese Academy of Medical Sciences and Peking Union Medical College, Beijing, China

**Keywords:** mucopolysaccharidosis type II, clinical characteristics, surgical history, genotypes, IDS gene variants

## Abstract

**Background:** Mucopolysaccharidosis Type II (MPS II) is a rare, progressive and ultimately fatal X-linked lysosomal storage disorder caused by mutations in the iduronate-2-sulfatase (IDS) gene. This report conducted a retrospective analysis to investigate the clinical characteristics, genotypes and management strategies in a large cohort of Chinese patients with MPS II.

**Methods:** In this study, we explored 130 Chinese patients with MPS II between September 2008 and April 2022. Clinical manifestations, auxiliary examination, IDS pathogenic gene variants and IDS enzyme activity, surgical history were analysed in the study.

**Results:** A total of 130 patients were enrolled and the mean age at diagnosis was 5 years old. This study found the most common symptoms in our patients were claw-like hands, followed by coarse facial features, birthmarks (Mongolian spot), delayed development, inguinal or umbilical hernia. The most commonly cardiac manifestations were valve abnormalities, which were mitral/tricuspid valve regurgitation (71.9%) and aortic/pulmonary valve regurgitation (36.8%). We had found 43 different IDS pathogenic gene variants in 55 patients, included 16 novel variants. The variants were concentrated in exon 9 (20% = 11/55), exon 3 (20% = 11/55) and exon 8 (15% = 8/55). A total of 50 patients (38.5%) underwent surgical treatment, receiving a total of 63 surgeries. The average age of first surgery was 2.6 years, and the majority of surgery (85.7%, 54/63) was operated before 4 years old. The most common and earliest surgery was hernia repair. Three patients were died of respiratory failure.

**Conclusion:** This study provided additional information on the clinical, cardiac ultrasound and surgical procedure in MPS II patients. Our study expanded the genotype spectrum of MPS II. Based on these data, characterization of MPS II patients group could be used to early diagnosis and treatment of the disease.

## 1 Introduction

Mucopolysaccharidosis type II (MPS II, Hunter Syndrome; OMIM 309900) is a rare, progressive and ultimately fatal X-linked disorder. It is caused by a deficiency of the enzyme iduronate-2-sulfatase (IDS; EC 3.1.6.13) due to the IDS gene variants (Xq28) (Clarke et al., 1992). Like all X-linked recessive disorders, MPS II affect males almost exclusively, although rare cases of MPS II in females do occur (Neufeld et al., 1977). The prevalence in European countries ranges from .13/100,000 to .71/100,000 (Khan et al., 2017), while the Asian countries is relatively higher. For example, the prevalence of live births in Korea and Japan are .74/100,000 and .84/100,000 respectively (Khan et al., 2017). MPS II accounted for approximately half (47.4%–58.0%) of all cases of mucopolysaccharidosis in Eastern Asian countries (Chen et al., 2016), compared with only 7.6% of mucopolysaccharidosis patients in the Netherlands and Portugal (Stapleton et al., 2017).

IDS deficiency leads to an accumulation of dermatan sulfate (DS) and heparan sulfate (HS) in multiple organs and tissues, resulting in progressive cell degradation and failure (Demydchuk et al., 2017). Organs implicated include the teeth, ear, nose and throat, heart and respiratory system, gastrointestinal tract and the musculoskeletal system, central nervous system (Stapleton et al., 2017). As a consequence, patients with MPS II present with multiple clinical manifestations that can include coarse facial features, hypertrophic tonsils and adenoids, claw-like hands or joint stiffness, scoliosis, otitis media, hepatosplenomegaly, abdominal or inguinal hernias (Wraith et al., 2008a; Martin et al., 2008). MPS II may also present with neurological manifestations (Wraith et al., 2008b). Patients typically have a normal appearance at birth afterwards symptoms beginning at 18 months to 4 years old (Scarpa et al., 2011). MPS II management has historically been limited to supportive and palliative treatments involving multiple clinical departments. The last decade has seen the emergence of enzyme replacement therapy (ERT), beginning with the approval of idursulfase in the United States in 2006. However, approval for MPS II ERT in China was only granted in 2019 (Kang et al., 2019). To date, more than 600 unique IDS pathogenic gene variants have been identified, with approximately half of these accounted for exonic single point variants (Human Gene Mutation Database (HGMD): IDS Gene: http://www.hgmd.cf.ac.uk).

Despite China’s large population, and the relatively high prevalence of MPS II in Eastern Asia, there had been few reports of the clinical characteristics, genotypes or management strategies for Chinese patients with MPS II. Most published data were limited to case reports or include relatively small patient numbers. There is no newborn screening for MPS II in mainland China. A deeper understanding of the clinical characteristics and current treatment approaches for MPS II in China would be highly valuable for improving disease awareness, rates of early detection and diagnosis, supporting early initiation of palliative/supportive care and ultimately improving patients’ quality of life. Therefore, we conducted a single-center retrospective study to investigate the clinical characteristics, genotypes and management strategies in a large cohort of Chinese patients with MPS II.

## 2 Materials and methods

### 2.1 Patients

This was a retrospective, single center, observational analysis of medical records for patients treated at Peking Union Medical College Hospital, Beijing, China, between September 2008 and April 2022. Eligible patients had a diagnosis of MPS II based on patient clinical presentation, laboratory examination, IDS enzyme activity in plasma and IDS gene analysis. Patients were also required to have medical records covering clinical presentation and treatment for MPS II. The study was approved by the hospital Ethics Committee and was conducted in accordance with the Helsinki Declaration ethical principles for medical research involving human subjects. Written informed consent was obtained from every patient or parents prior to inclusion in the study.

### 2.2 Clinical assessments

Clinical manifestations were collected by medical records. Cardiac ultrasound was used to assess for cardiac manifestations of MPS II. Brain MRI was used to evaluate cranial manifestations of MPS II.

### 2.3 Enzyme activity testing

IDS activity levels in plasma were determined by fluorimetric enzyme assay using 4-methylbelliferone-labelled iduronate-2-sulfate as a substrate for IDS, as previously described (Voznyi et al., 2001). The assays were performed at the Department of Genetics laboratory, Peking Union Medical College Hospital. The normal value was 240.2–668.2 nmol/4 h/mL. MPS II could be diagnosed when the IDS enzyme activity result was less than 20 nmol/4 h/mL.

### 2.4 IDS genetic analysis

Peripheral blood was collected in EDTA tubes and genomic DNA was isolated using the QIAamp DNA Blood Midi Kit (Qiagen, Germany). DNA concentration was quantitatively isolated by NanoDrop 2000 (Thermo Fisher Scientific, MA). The exonic regions and exon-intron boundaries of the IDS gene were then amplified by PCR and sequenced using the Sanger technique and standard protocols (Kosuga et al., 2016).

### 2.5 *In silico* analysis

A variety of pathogenicity prediction tools were used to predict the pathogenicity potential of the novel pathogenic gene variants. Mutation Taster (http://www.mutationtaster.org), SIFT/PROVEAN (http://provean.jcvi.org) and PolyPhen-2 (http://genetics.bwh.harvard.edu/pph2) web software.

### 2.6 Statistics

The quantitative data were summarized by means and standard deviations (SDs). Pathogenic gene variant types and clinical signs were represented by frequency distribution. Descriptive statistics were used to summarize data. All analyses were conducted using SPSS v19.0 (IBM, United States).

## 3 Results

### 3.1 Patients

In total, 269 patients were diagnosed with MPS II in our hospital during the study period of September 2008 to April 2022. Among them, 130 patients from 119 families had suitable medical records available and were included in the study analysis. All included patients were male and 11 had a known family history of MPS II. The mean age at diagnosis was 5 years old ±2.6 (range: 2 months to 22 years old).

### 3.2 Clinical manifestations

The most common clinical manifestations were musculoskeletal and dermatologic abnormalities. The onset age of different clinical manifestations in MPS II patients was shown in Table 1. 98.0% (99/101) patients had claw-like hands (joint stiffness), most of these patients (63.4%, 64/101) occurred between 2 and 6 years old. 97.9% (97/99) patients had coarse facial features, which also commonly occurred in patients aged 2–6 (67.6%, 67/99). In addition, 95.8% (91/95) patients had birthmarks (Mongolian spot), 79.6% (78/98) had delayed development or growth, 69.4% (68/98) had inguinal or umbilical hernia, 59.4% (60/101) had sleep apnoea, 37.3% (38/102) had spine deformities, and 25.5% (25/98) patients had deafness. It could be seen in Table 1 that the onset of all kinds of MPS II symptoms generally occurred between 2 and 6 years old.

**TABLE 1:** Patient age at onset of clinical manifestations of MPS II. Percentage (Number).

	Claw-like hands	Coarse facial	Delayed development	Hernia	Sleep apnoea	Spine deformities	Deafness
<2 years old	18.8% (19)	13.1% (13)	11.2% (11)	8.1% (8)	3.9% (4)	6.9% (7)	2.0% (2)
2–4 years old	33.7% (34)	36.3% (36)	31.6% (31)	21.4% (21)	21.8% (22)	10.8% (11)	9.2% (9)
4–6 years old	29.7% (30)	31.3% (31)	23.4% (23)	28.6% (28)	20.8% (21)	13.7% (14)	6.1% (6)
6–8 years old	5.9% (6)	6.0% (6)	6.1% (6)	4.1% (4)	7.9% (8)	1.0% (1)	3.1% (3)
>8 years old	9.9% (10)	11.1% (11)	7.1% (7)	7.1% (7)	4.9% (5)	4.9% (5)	5.1% (5)
Positive	98.0% (99)	97.9% (97)	79.6% (78)	69.4% (68)	59.4% (60)	37.3% (38)	25.5% (25)
Total	100% (101)	100% (99)	100% (98)	100% (98)	100% (101)	100% (102)	100% (98)

### 3.3 Cardiac ultrasound and brain MRI

**FIGURE 1:**
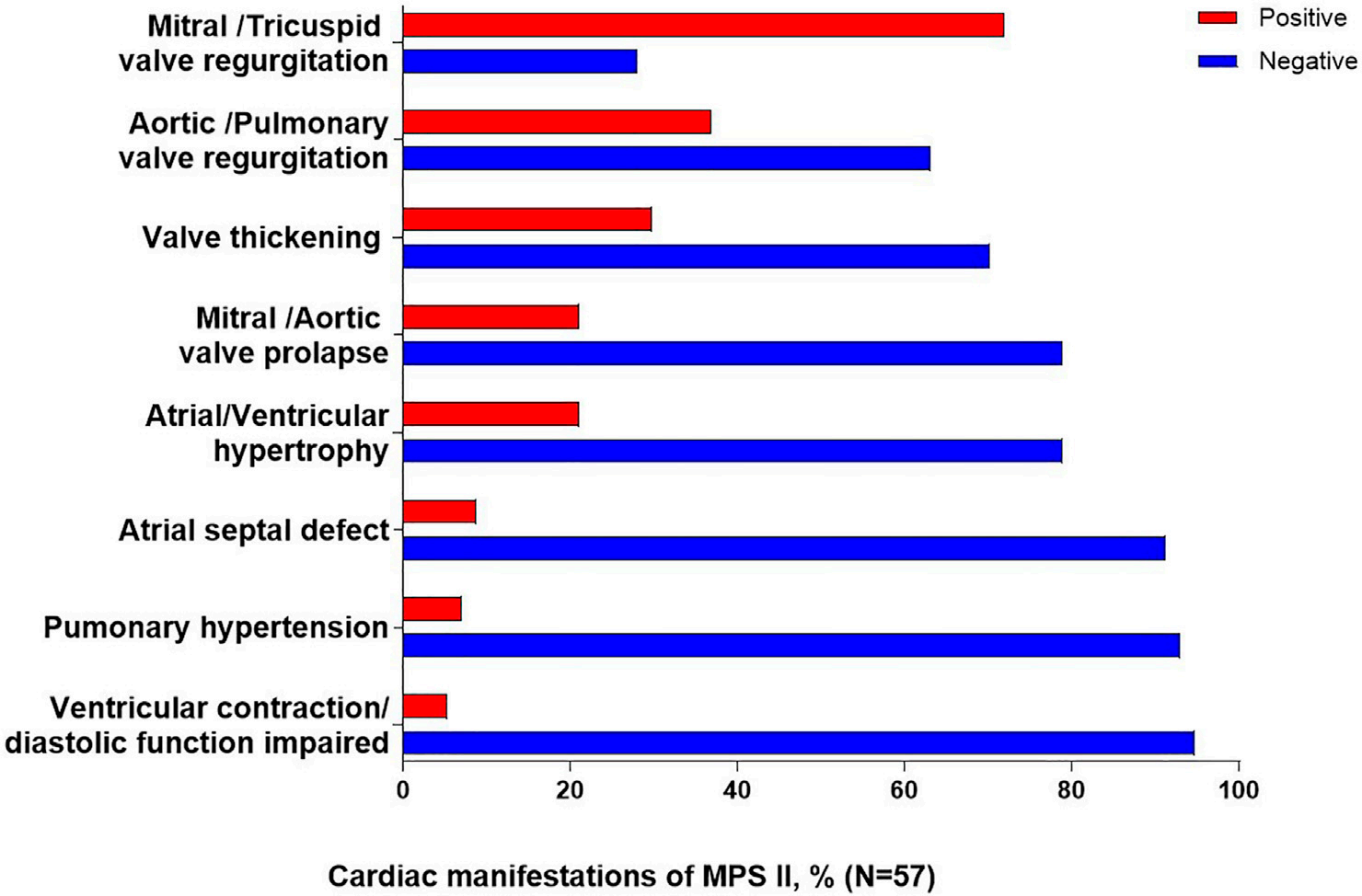
Cardiac clinical manifestations of MPS II. Percentages are calculated as a proportion of the total patients who underwent cardiac ultrasound (N = 57).

A total of 57 patients underwent cardiac ultrasound, and of these patients, valve abnormalities were the most commonly observed cardiac manifestation of MPS II (Figure 1). The most common specific manifestations observed were mitral/tricuspid valve regurgitation (41/57; 71.9%), aortic/pulmonary valve regurgitation (21/57; 36.8%), valve thickening (17/57; 29.8%), mitral/aortic valve prolapse (12/57; 21.1%) and atrial/ventricular hypertrophy (12/57; 21.1%).

A total of 30 patients checked brain MRI, of which 12 patients had abnormal results and 18 patients had normal results. Of the 12 patients with abnormalities detected on brain MRI imaging, seven patients had cystic -like brain cystic sample changes caused by widened vascular space, two patients had diffuse brain atrophy, three patients had expansion of the ventricle and hydrocephalus.

### 3.4 IDS pathogenic gene variants

IDS gene sequencing data was available for 59 patients, of which four patients had no pathogenic variant detected by IDS gene analysis. The IDS pathogenic gene variants of 55 patients were summarised in Table 2 (NM_000202, NM_001166550, and NM_006123). A total of 43 different variants types were detected. Of the 43 variants, 16 (37%) novel variants were detected: six exonic point variants (c.260G>C, c.261C>A, c.325T>G, c.456C>G, c.1432G>C, and c.1453A>T), three small insertions (c.438_439insTT, c.1489_1490insT, and c.1269dupC), five small deletions (c.41delG, c.314_339del, c.449delC, c.481_486del, and c.794delA), one small indels (c.801_806delinsTGGC), one duplication (Exon8 duplication).

**TABLE 2:** The IDS variants genetype in Chinese patients with MPS II (*n* = 55).

Category	Variant	Consequence	No. of occurrences	Novel variant
Exonic point variant	c.1122C>T	p.G374G (loss of 20 aa)	4	—
	c.262C>T	p.R88C	3	—
	c.514C>T	p.R172X	3	—
	c.934G>T	p.G312C	2	—
	c.998C>T	p.S333L	2	—
	c.1402C>T	p.R468W	2	—
	c.22C>T	p.R8X	1	—
	c.122T>C	p.L41P	1	—
	c.257C>T	p.P86L	1	—
	c.260G>C	p.S87T	1	Novel
	c.261C>A	p.S87R	1	Novel
	c.309C>A	p.Y103X	1	—
	c.325T>G	p.W109G	1	Novel
	c.404A>G	p.K135R	1	—
	c.456C>G	p.S152R	1	Novel
	c.1025A>C	p.H342P	1	—
	c.1030G>A	p.E344K	1	—
	c.1034G>A	p.W345X	1	—
	c.1327C>T	p.R443X	1	—
	c.1425G>A	p.W475X	1	—
	c.1432G>C	p.D478H	1	Novel
	c.1453A>T	p.I485L	1	Novel
	c.1562A>T	p.E521V	1	—
	c.1568A>G	p.Y523C	1	—
Gross deletion	EX1_9del		1	—
	EX1_6del		1	—
	EX7del		1	—
	EX9del		1	—
Small deletion	c.449delC	p.P150R	2	Novel
	c.596_599delAACA	p.K199Rfs*13	2	—
	c.41delG	p.G14Vfs*4	1	Novel
	c.314_339del	p.F105Wfs*31	1	Novel
	c.481_486del	p.161_162del	1	Novel
	c.794delA	p.N265Tfs*15	1	Novel
Small insertion	c.394_395insGT	p.S132Cfs*82	1	—
	c.438_439insTT	p.D147Lfs*67	1	Novel
	c.1269dupC	p.V424Rfs*7	1	Novel
	c.1489_1490insT	p.Y497Lfs*2	1	Novel
Splicing	c.419–2A>G		1	—
	c.507 + 1G>A		1	—
	c.1006 + 1G>A		1	—
Duplication	Exon8 duplication		1	Novel
Small indels	c.801_806delinsTGGC	p.W267Cfs*74	1	Novel

Twenty-four exonic point variants were detected in 34 patients which were the most frequent type of variant (61.8% = 34/55). The most common point variant was c.1122C>T, which was detected in 7.3% (4/55) of patients. The second most frequent variant was c.262C>T and c.514C>T, which were detected in 5.5% (3/55) of patients separately. All three of above variants had been detected previously in other studies of patients with MPS II. The other variant types of patients included four gross deletions, eight small deletions, four small insertions, three splicings, one duplication, one small indels variant.

**FIGURE 2:**
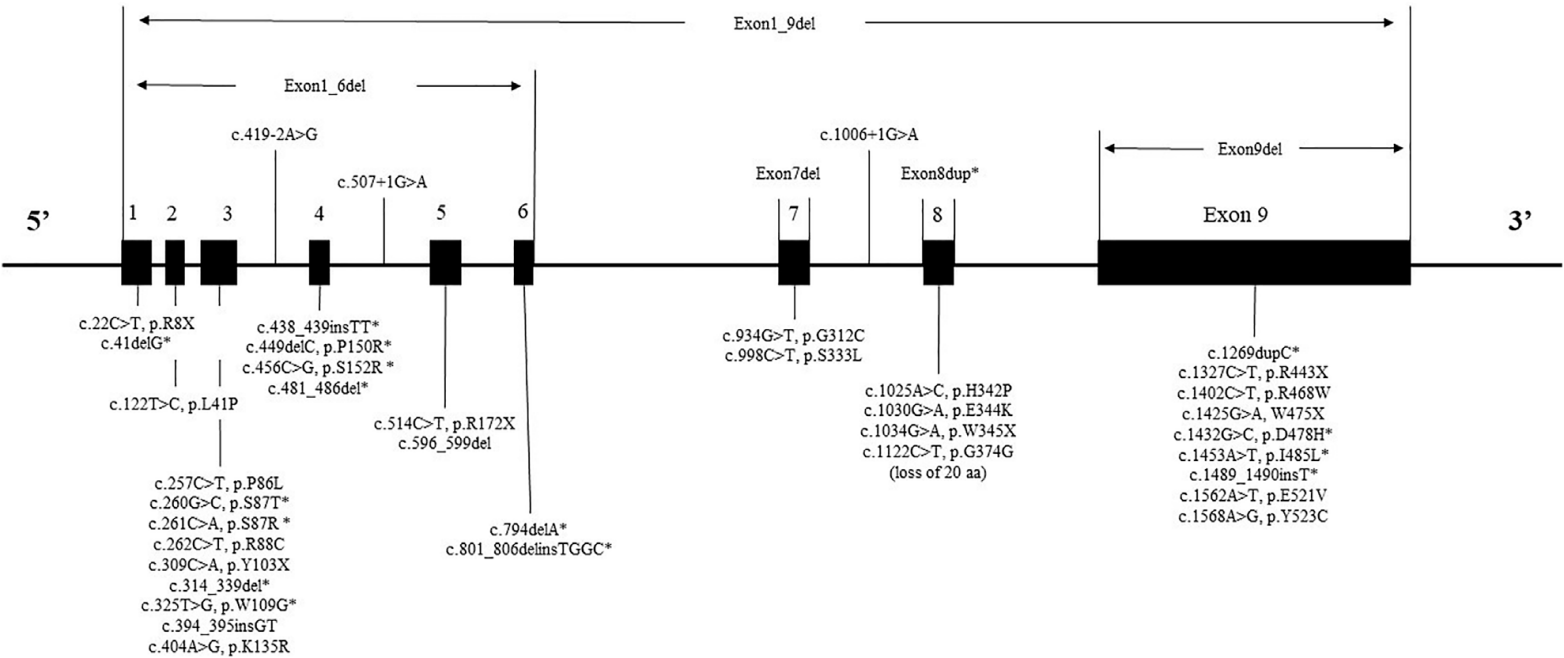
The mutant spectrum of IDS. * represents a novel variant.

The mutant spectrum of IDS could be seen in Figure 2. While variants were found in all exons, the variants of patients were concentrated in exon 9 (20% = 11/55), exon 3 (20% = 11/55) and exon 8 (15% = 8/55). The IDS gene variant had no variant hotspots, but had variant “hot exon.”

### 3.5 IDS enzyme activity

Data on plasma IDS enzyme activity was available for 100 patients (76.9%). IDS activity levels ranged from .0 to 11.1 nmol/4 h/mL. The mean enzyme activity was .72 ± .14 nmol/4 h/mL, which was below our institution diagnosed value 20 nmol/4 h/mL.

### 3.6 *In silico* analysis of the novel variants

All of the novel variants in the IDS gene on protein function were indicated deleterious by the pathogenicity prediction tool Mutation Taster. Meanwhile, the novel exonic point variants were predicted damaging by the Polyphen-2 and SIFT/PROVEAN web software. This was also demonstrated by the reduced IDS enzyme activity.

### 3.7 Treatment

Treatment was generally supportive and palliative in nature, in line with treatment practice in China over the study period. Two patients underwent bone marrow transplant. A total of 50 patients (38.5%) underwent surgical treatment, receiving a total of 63 surgeries. Eight patients (6.2%) underwent ≥2 surgeries and the highest number of surgeries received by a single patient was five (including three hernia surgeries, surgeries for the adenoids and correction of congenital megacolon).

The most common palliative surgery was hernia repair (three cases of umbilical hernia and 41 inguinal hernias), which accounted for 69.8% of all surgeries received by patients, followed by 13 cases of adenoidectomy or tonsillectomy, which accounted for 20.6% of all surgeries received. Other surgical procedures included: one eye surgery, two nasal polyp removal surgeries, two intestinal surgeries and one scoliosis correction.

**FIGURE 3:**
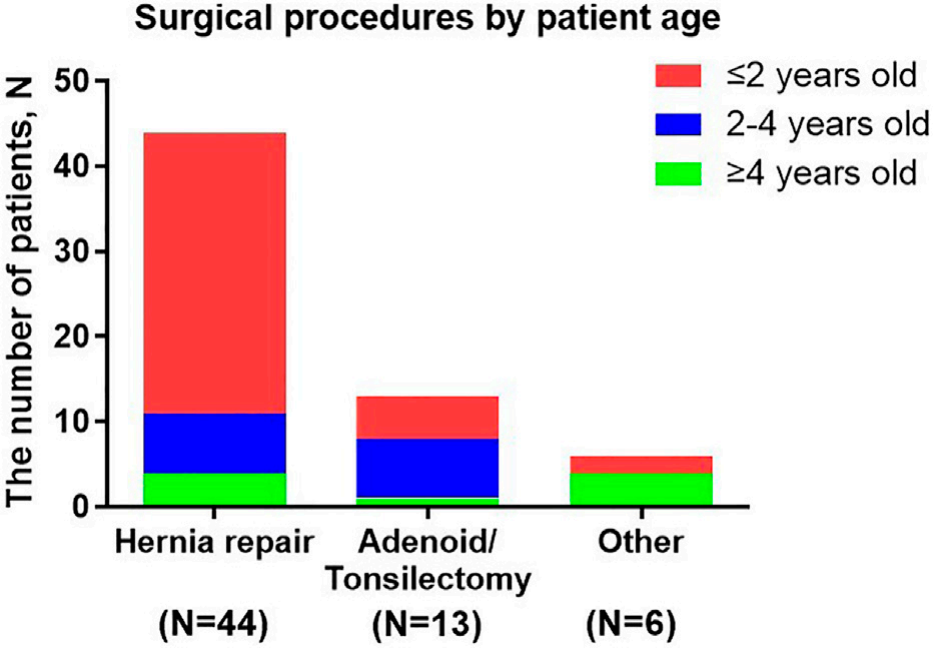
Surgical procedures by patient age. Other surgical procedures included: eye surgery, nasal polyp removal, intestinal surgery and scoliosis correction.

The average age of patients on receiving their first surgery was 2.6 years old, and the majority of surgery (85.7%, 54/63) was operated before 4 years old (Figure 3). The earliest operations were hernia repairs (at a median age of 1.8 years). 75% hernia repairs were operated before 2 years old and 90.9% were operated before 4 years old.

### 3.8 Morbidity and mortality

Two patients, aged 11 years and 15 years, respectively, were wheelchair-bound and one 11-year-old patient was unable to eat independently. Three patients were now known to died, aged 10, 11, and 13 years, respectively. The cause of death in all three cases was respiratory failure.

## 4 Discussion

This was a large scale analysis of clinical and molecular characterizations of Chinese patients with Mucopolysaccharidosis Type II. The MPS II was one of the diseases that involved multiple systems (Ullah et al., 2017; Ullah et al., 2018). The most common symptoms in our study were claw-like hands or joint stiffness, followed by coarse facial features, birthmarks (Mongolian spot), delayed growth or development, inguinal or umbilical hernia, sleep apnoea, spine deformities, deafness. The onset of MPS II symptoms generally occurred between the age of 2 and 6 years. More than one-third of the patients had surgery, the most common and earliest surgery was hernia repair. We had found 43 different IDS gene variants in 55 patients, included 16 novel variants. The variants were concentrated in exon 9, exon 3 and exon 8.

In China, MPS II was the most frequently occurring MPS (Fang et al., 2022). The median age at diagnosis in the Chinses population in this analysis was a little older than those reported previously in the global Hunter Outcome Survey (Wraith et al., 2008a) (5.0 years vs. 3.5 years) (HOS was a global multicentre registry of MPS II patients). Consistent with other findings, facial features, claw-like hands and birthmarks were the most common clinical manifestations of MPS II. The incidence rate of signs and symptoms in our study was almost same with other studies (Lin et al., 2018), claw hands (98.0% vs. 98.2%), facial features (97.9% vs. 100%).

The accumulation of GAG in cardiac valves and myocardium lead to valvular defects and cardiomyopathy (Costa et al., 2017). The mitral or aortic leaflet thickening and calcification could cause valvular stenosis and regurgitation. The deformities in cardiac structures could cause cardiac dysfunction, which could significantly increase the morbidity and mortality of affected patients (Chkioua et al., 2020). In the other study, respiratory failure was the primary cause of death (56%), followed by cardiac failure (18%) (Lin et al., 2016).

In our study, valve abnormalities were the most commonly observed cardiac manifestation of MPS II. The most common specific manifestations observed were mitral/tricuspid valve regurgitation (71.9%), aortic/pulmonary valve regurgitation (36.8%), in agreement with the report by Lin et al. (2021). Therefore, echocardiography should be checked regularly (Scarpa et al., 2011).

IDS gene sequencing data was available for 55 patients and 43 different variants types were detected. In this study, sixteen novel variants (37%) were detected, the rate of discovery of novel genes was consistent with another study from China (32.7%) (Zhang et al., 2019). It had been reported that, variants tend to be more frequent in exons 3, 7, 8, and 9 (Rathmann et al., 1996; Agrawal et al., 2022). As expected, in our sample, the highest frequency of variant exons were 9, 3, and 8. Consistent with data from other Chinese study, exons 9 and 3 had comparatively more variants (Zhang et al., 2011).

In our study, the most common variant identified was c.1122C>T, in codon 374, constituting about 7.3% (4/55) of all variants. This was also the most frequent variant of the IDS gene in Spain, Portugal and Latin American patients, which more than 30 patients had been reported (Brusius-Facchin et al., 2014). Other two studies from China reported the codon 468 position could be a “hot” spot, which five patients showed variants from 63 patients and 38 patients, respectively (Zhang et al., 2011; Zhang et al., 2019). In our study, we found two patients had variants with p.R468W.

This analysis was one of the largest studies about surgical records in Chinese MPS II patients. A total of 50 patients (38.5%) in this analysis had undergone surgical treatment, while the occurrence rate of surgical treatment in HOS was more than three-quarters (Mendelsohn et al., 2010). The age at first surgery in Chinese patients was almost the same as the global HOS population (2.6 years) and the vast majority of surgeries was undergone before 4 years old. The earliest operations to be performed were hernia repairs. However, some surgeries prevalence in our study had a little different from the global HOS population (Mendelsohn et al., 2010): hernia repair (69.8% vs. 50.1%), adenoidectomy or tonsillectomy (20.6% vs. 85.0%). Clinicians and caregivers in China may had different perceptions of the need for MPS II surgery compared with other countries, which might explain these differences in the frequency of surgery among Chinese patients and HOS population. The other studies showed tympanostomy (placement of ear-drum ventilating tubes) were commonly performed operations and the incidence rate was 51.4% and 27.9% (Mendelsohn et al., 2010; Lin et al., 2018). However, few patients in our study performed this surgery. It indicated that hearing screening at follow-up was important.

The knowledge gained from this study broadens the list of indicators of MPS II to include early and repeated surgical intervention. Some of the symptoms associated with hernias and adenoid/tonsilectomy might suggest that the patient had MPS II, which could be helpful for early diagnosis of the disease. Early diagnosis permitted early initiation of ERT, which could decrease complications, particularly before organ damage becoming irreversible (Scarpa et al., 2011). Prompt diagnosis might also mean that complications during surgical intervention could be avoided.

Usually, the main criterion for severity relies on the degree of intellectual disability. But intellectual disability was not the main cause of death in MPS II, most patients died of respiratory failure and cardiac failure in our study and other studies (Lin et al., 2016). The intellectual disability could reduce the quality of life, but it did not affect longevity. We believed that cardiopulmonary function was the main cause of death in the disease, therefore, it was more appropriate to distinguish between mild and severe subtype by whether cardiopulmonary function was affected or not. According to the cardiopulmonary function, a standard scoring system was recommended, which could evaluate the patient’s survival time and prognosis.

Since MPS II was an X-linked genetic disease, molecular diagnosis was important for carrier detection and prenatal diagnosis. The other studies identified carrier status in 70%–72% of the mothers of the probands (Agrawal et al., 2022). Parenteral diagnosis could play a major role in reducing the burden of such severe disorders.

It was important to consider the limitations of this study. As a retrospective and single-center study, some medical records were missing and not available at the time of this study. This study did not analysis the genotype–phenotype correlations in MPS II. MPS II genetic heterogeneity represents a major difficulty in establishing genotype–phenotype correlations. The problem was complicated by the lack of a standardized scoring index of severity in clinical evaluation and the comparison of clinical phenotypes was difficult and subjective. Therefore, a greater number of patients and longer follow-up would be needed to investigate.

Despite these limitations, this was one of the largest studies of clinical presentations and surgical histories in Chinese patients. By continuously recording and analyzing clinical and laboratory data of various patients, we would deepen our understanding of MPS II in China. Understanding surgical history patterns in patients with MPS II would help improve diagnosis, disease management, prospective guidance, and quality of life. Analysis of longitudinal surgical data from a large number of Chinese patients provided unique insights into the surgical history of MPS II patients. Furthermore, the disease was complex, and management was often challenging and required a multidisciplinary approach.

## 5 Conclusion

In conclusion, this paper provided additional information on the clinical, cardiac ultrasound and surgical procedure in MPS II patients. This analysis found that claw-hands, birthmarks and facial features were the most common symptoms. The valve abnormalities were the most commonly cardiac manifestation of MPS II, which were mitral/tricuspid valve regurgitation and aortic/pulmonary valve regurgitation. Our study expanded the spectrum of genotype of MPS II. We had found 43 different IDS gene variants in 55 patients, included 16 novel variants. The variants were concentrated in exon 9, 3 and 8. A total of 38.5% of the patients underwent a surgical procedure. The most common and earliest surgery was hernia repair. Our study expanded the genotype spectrum of MPS II. Based on these data, characterization of MPS II patients group could be used to early diagnosis and treatment of the disease.

## Data Availability

The original contributions presented in the study are included in the article/supplementary material, further inquiries can be directed to the corresponding author.

## Abbreviations

DS, Dermatan sulfate; ERT, Enzyme replacement therapy; HS, Heparan sulfate; HGMD, Human Gene Mutation Database; HOS, Hunter Outcome Survey; IDS, Iduronate-2-sulfatase; MPS II, Mucopolysaccharidosis Type II; NCBI, National Center for Biotechnology Information.
